# Happy Hour: The association between trait hedonic capacity and motivation to drink alcohol

**DOI:** 10.1016/j.abrep.2024.100537

**Published:** 2024-03-05

**Authors:** Daniela Becker, Katharina Bernecker

**Affiliations:** a Leibniz-Institut für Wissensmedien, Schleichstraße 6, 72076 Tübingen, Germany; b Behavioural Science Institute, Radboud University, Thomas van Aquinostraat 4, 6525 GD Nijmegen, The Netherlands; c University of Zurich, Allgemeine Psychologie (Motivation), Binzmühlestrasse 14/Box6, 050 Zürich, Switzerland; d URPP Dynamics of Healthy Aging, University of Zurich, Switzerland

**Keywords:** Alcohol use, Hedonic capacity, Motivation to drink, Self-regulation, Stress

## Abstract

The (over)consumption of alcohol and other addictive substances is often conceptualized as a problem of low self-control (i.e., people’s inability to inhibit unwanted impulses). According to that view, people drink because they cannot resist. In the present studies, we approached this from a different perspective and tested whether alcohol consumption might also be a problem of low hedonic capacity (i.e., people’s inability to experience pleasure and relaxation, often due to intrusive thoughts). According to that view, people drink because it helps them enjoy or cope with negative thoughts or emotions. In two studies among individuals at low risk of harmful alcohol use (e.g., AUDIT < 7) we consistently found that trait hedonic capacity was unrelated to alcohol consumption but negatively related to coping motivation (drinking alcohol to cope with negative thoughts and feelings; Study 1: *N* = 348; Study 2: *N* = 302, preregistered). Exploratory analyses in study 2 (conducted during the COVID-19 pandemic) also showed that people with low, but not high, trait hedonic capacity drank more alcohol in response to stress. Our findings are in line with the notion that people’s drinking motivation and behavior might not only be a problem of poor self-control but also of low trait hedonic capacity. They align with a new direction in addiction prevention and treatment research, which explores ways to help people to seek out and savor hedonic experiences from non-drug related reinforcers (e.g., engaging in leisure activities).

## Highlights

• Low hedonic capacity is related to a higher motivation to drink alcohol out of coping motives.
• Drinking out of coping motives is related to more alcohol consumption.
• People with low hedonic capacity seem to drink more as a response to stress.
• Enhancing hedonic capacity might have preventive and interventive value in the addiction domain.

## 1 Introduction

Many people know the situation of coming home from a stressful day, hoping to relax. It is not uncommon that people in those situations pour themselves an alcoholic drink. Whereas the occasional alcoholic drink can be fine, the repeated occurrence of this behavior can, over time, turn into an addiction. In fact, recent reports of alcohol use in Europe suggest that one in twelve people drink alcohol on a daily basis and roughly one out of three drink every week (Eurostat, 2019). Although men still consume more alcohol than women (Eurostat, 2019), women’s alcohol use has been increasing over the last years in the US (White, 2020). That is a problem because alcohol use represents one of the greatest reasons for premature death and ill health (WHO, 2019). The World Health Organization made it, therefore, a public health priority to reduce harmful alcohol use by 2030 (WHO, 2019). The aim of the current study is to extend our knowledge on the factors related to alcohol use in the general public. Specifically, we investigate the relationship between trait level differences in people’s general ability to successfully experience pleasure or enjoyment during a hedonic activity (e.g., relaxing on the sofa), drinking motives and actual alcohol use.

### 1.1 Hedonic capacity and (motivation for) alcohol use

Harmful alcohol use is often described from a dual-process perspective, according to which there is a disbalance between a strong impulse (i.e., craving) for the substance, and a reduced ability to control those impulses (De Wit, 2009, Goldstein and Volkow, 2011, Wiers and Stacy, 2006, Wiers et al., 2010). Accordingly, researchers have tried to tackle harmful drinking by enhancing people’s control over their impulses through, for example, inhibition training (Houben et al., 2011, Wiers et al., 2013). Those impulses can be direct behaviors (e.g., reaching for the alcohol) but also recurring thoughts about alcohol (e.g., craving). In the present research we approach alcohol use from a different perspective: Instead of conceptualizing it as a lack of people’s capacity to control their impulses (i.e., self-control), we argue that it can also stem from a lack of people’s trait *hedonic capacity* (Bernecker & Becker, 2021; cf. Meehl, 1975). Trait hedonic capacity describes the extent to which people successfully experience pleasure or enjoyment during a hedonic activity (e.g., relaxing on the sofa). People high in hedonic capacity report higher levels of positive affect during daily hedonic activities (e.g., walking in nature, socializing) but also higher levels of overall well-being and life satisfaction (Bernecker & Becker, 2021). One of the factors that has been found to lower hedonic capacity (on the trait and situational level) is the experience of intrusive thoughts, for example about conflicting goals (e.g., thoughts about work keep you from relaxing; Bernecker & Becker, 2021). In the context of drinking, we propose that people’s level of hedonic capacity may be related to their alcohol use because alcohol could help to either enhance positive affect or reduce intrusive thoughts. In other words, we argue that people do not only drink because they may lack the control necessary to inhibit the impulse, but also because they may not otherwise find the enjoyment and distraction they seek.

Our conceptualization is in line with research on drinking motives, which broadly distinguishes between the motivation to enhance positive affect (enhancement motive) and the motivation to reduce negative affect (coping motive; Cooper et al., 2015, Cox and Klinger, 1988). Studies show that both motives are related to drinking behavior, but in different ways. For example, even though both motives are related to increased alcohol use in general, coping motives are more strongly and reliably associated with problematic drinking behavior (e.g., binge drinking; problems with partner due to alcohol; Cooper et al., 2015). People who drink out of coping motives also show a stronger attentional bias for alcohol (vs. control) stimuli when stressed (Field & Quigley, 2009), and explicitly endorse the belief that alcohol can help cope with negative mood states (Birch et al., 2004). This suggests that for them alcohol represents a central mean to cope with stress and negative affective states (McHugh & Kneeland, 2019).

That a lack of positive affect may predispose to harmful drinking is also evident from clinical studies linking anhedonia (symptomatic inability to experience pleasure in response to pleasant stimuli; Meehl, 1975, Snaith, 1993) to the risk of substance use and escalation (for a review see Destoop et al., 2019). Given the neuropsychological properties of addictive substances, they can stimulate brain reward circuits and thus boost affective experiences. Over time and with chronic substance use, people become more sensitive to substance-related cues as they have gained strong reward properties. This translates into enhanced reward seeking behavior (‘wanting’) which can motivate enhanced substance use but leaves ‘liking’ of the substance unchanged or even blunted (Berridge and Robinson, 2016, Garland, 2021). A large corpus of neuropsychological evidence supports this notion by showing changes in mesolimbic and dopaminergic brain systems reflecting substance-related ‘wanting’ responses (for an overview see Thomsen et al., 2015).

However, most of the studies relating anhedonia to substance use are conducted in clinical samples, investigating comorbidities across psychiatric disorders. Trait hedonic capacity on the other hand, taps into people’s more general self-regulatory ability to successfully engage in hedonic goal pursuit and is unrelated to anhedonia in non-clinical populations (Bernecker & Becker, 2021). By investigating the relationship between trait hedonic capacity and motives to drink and alcohol use, we combine recent insights from self-regulation research and the clinical domain with the aim to get a broader picture of the role successful self-regulation plays in alcohol consumption (Becker & Bernecker, 2023).

### 1.2 The present studies

The present studies investigate the relationship between people’s trait hedonic capacity, people’s alcohol use and their motivation to drink (coping vs. enhancement) in a non-clinical sample at low risk of harmful drinking. Study 1 explored those relationships in a representative sample. Based on the literature, we expected that trait hedonic capacity is related to people’s motivation to drink (lower trait hedonic capacity is associated with stronger motivation), and possibly also to more frequent alcohol use. Not all of those expectations were confirmed. Study 2 was set up as a replication study with validated measurement instruments for which we preregistered the findings of study 1.

Data (study 1 and 2) and preregistration (study 2) are openly available here (https://osf.io/bjp8a/). Both studies have approval from the local ethics committee.

## 2 Study 1

### 2.1 Method

#### 2.1.1 Participants

We recruited 348 English-speaking participants (211 females, 136 males, 1 gender queer/nonconforming; *M*_age_ = 32.96, *SD* = 11.33; range 18–77 years) via the online platform Prolific.com. The overall study took 40 min and participation was compensated with £ 5. About 50 % of the sample indicated to work full-time, 21 % worked part-time, 14 % was registered as a student, 11 % was looking for work and 4 % were retired.

#### 2.1.2 Materials and procedure

After giving informed consent, participants completed several questionnaires, which are reported elsewhere (see Bernecker and Becker, 2021). The current exploratory study was the final part of a larger study (reported as Study 2A in Bernecker and Becker (2021). Final *N* of this exploratory study deviated from the final *N* reported in Bernecker and Becker (2021) because 2 people indicated after the drug-use related questions that they do not want their data to be analyzed and reported.

***Trait hedonic capacity.*** Participants filled in the 10-item measure of Trait Hedonic Capacity (THC, Bernecker & Becker, 2021), which consists of two subscales: five items measuring hedonic success (e.g., “I am good at pursuing my desires”) and five items measuring intrusive thoughts (e.g., “I often think about my duties even while I am enjoying a good moment”). Questions were answered on 5-point Likert scales, with higher scores indicating higher applicability (1 = *not at all* – 5 *= very much*). An mean score was calculated, with higher scores indicating higher levels of trait hedonic capacity (Cronbach’s α = 0.83). We also created a mean score for both subscales (hedonic success: Cronbach’s α = 0.83; intrusive thoughts: Cronbach’s α = 0.82).

***Alcohol use and drinking motives*** Alcohol use was measured with one item (“How often do you drink alcohol”) and participants could indicate their answer on a 5 point scale (*1 = never; 2 = monthly or less; 3 = 2*–*4 times per months; 4 = 2*–*3 times per week; 5 = 4 or more times a week*). Motivation to drink was measured with 6 discrete motives adapted from the Drinking Motive Questionnaire Revised (DMQ-R, Cooper, 1994): “When you drink alcohol, to what extent do you drink because of the following reasons: social reasons; recovery from stress; relaxation; switching off; fun; habit?”. All questions were answered on a 5-point Likert scale, ranging from *never or seldom (1)* to *very often (5).* At the end of the questionnaire, participants were thanked, debriefed and compensated.

#### 2.1.3 Analysis plan

Given the exploratory nature of study 1 we only conducted bivariate correlations between the variables of interest.

### 2.2 Results and discussion

Regarding alcohol use, there was no correlation with THC, but the intrusive thoughts subscale showed a small positive correlation (see Table 1 for all correlations). Regarding drinking motives, there was only one significant negative correlation, namely between the whole THC scale and stress coping. That correlation with stress coping was also found for the subscale intrusive thoughts. That implies that struggling to have pleasurable hedonic experiences, often because of intrusive thoughts, is related to using alcohol for coping. Finally, all drinking motives apart from the social motives were related to alcohol use. Stress coping being the motive that had the second biggest correlation coefficient, after habits.

**Table 1:** Zero-Order Pearson Correlations for Trait Hedonic Capacity and Six Drinking Motives in Study 1.

Variable	*M*	*SD*	1	2	3	4	5	6	7	8	9	10
1. THC	2.87	0.69	–									
2. THC HS	3.31	0.83	0.83*** [0.79, 0.86]	–								
3. THC IT	3.58	0.84	−0.83*** [−0.86, −0.79]	−0.38*** [−0.46, −0.28]	–							
4. Alcohol use	2.72	1.26	−0.04 [−0.15, 0.06]	0.04 [−0.06, 0.15]	0.11* [0.01, 0.21]	–						
5. Social reasons	3.79	1.19	0.04 [−0.08, 0.16]	0.06 [−0.06, 0.18]	−0.01 [−0.12, 0.11]	−0.04 [−0.16, 0.07]	–					
6. Stress coping	2.51	1.31	−0.19** [−0.30, −0.07]	−0.06 [−0.18, 0.06]	0.24*** [0.13, 0.35]	0.44*** [0.34, 0.53]	−0.13* [−0.24, −0.01]	–				
7. Relaxation	3.22	1.28	−0.01 [−0.12, 0.11]	0.10 [−0.02, 0.21]	0.11 [−0.01, 0.22]	0.42*** [0.32, 0.52]	−0.11 [−0.23, 0.01]	0.62*** [0.54, 0.69]	–			
8. Switching off	2.96	1.35	−0.07 [−0.18, 0.05]	−0.01 [−0.13, 0.11]	0.10 [−0.02, 0.22]	0.38*** [0.28, 0.48]	−0.08 [−0.20, 0.04]	0.66*** [0.58, 0.72]	0.75*** [0.69, 0.80]	–		
9. Fun	3.61	1.17	−0.02 [−0.14, 0.10]	0.07 [−0.05, 0.18]	0.10 [−0.02, 0.21]	0.17** [0.05, 0.28]	0.33*** [0.22, 0.43]	0.13* [0.01, 0.24]	0.25*** [0.13, 0.35]	0.24*** [0.13, 0.35]	–	
10. Habit	2.07	1.36	−0.02 [−0.13, 0.10]	0.13* [0.01, 0.24]	0.15* [0.03, 0.26]	0.62*** [0.54, 0.69]	−0.03 [−0.14, 0.10]	0.56*** [0.47, 0.63]	0.51*** [0.42, 0.59]	0.52*** [0.43, 0.60]	0.19** [0.07, 0.30]	–

*Note*. THC = Trait hedonic capacity. THC HS = trait hedonic capacity subscale hedonic success. THC IT = trait hedonic capacity subscale intrusive thoughts. Numbers in brackets refer to 95 % confidence interval of the correlation coefficient. Significance levels: *** means *p* < .001; ** means *p* < .01; * means *p* < .05.

## 3 Study 2

Study 2 is a replication of the exploratory findings in study 1 using established measures of alcohol use and drinking motives. We preregistered two hypotheses (https://aspredicted.org/ic7vu.pdf):

H1: Trait hedonic capacity is unrelated to alcohol use.
H2: Trait hedonic capacity is negatively related to people’s coping motives, but unrelated to enhancement motives.

Given that we expected two null-effects, we complemented the preregistered frequentist analyses with Bayesian analyses. Additionally, we explored the role of stress in the hypothesized relations.

### 3.1 Method

#### 3.1.1 Participants

Based on an a-priori power analysis we planned to recruit 133 participants (80 % power, α = 5 %, medium effect size *r* = −0.20). In total, *N* = 480 people clicked on the link to participate in the study. After exclusion (according to preregistered criteria) the final sample consisted of *n* = 302 participants (*M*_age_ = 23.53, *SD* = 4.28; 227 female, 72 male, 2 no specification, 1 missing; 96 % students). Participants were recruited via the University of Tübingen mailing list and they could win a € 10 voucher. Data was collected in June 2020, which coincided with the first Covid-19 lockdown.

#### 3.1.2 Materials and procedure

After giving informed consent and demographic information, participants completed the measures in the following order.

***Trait hedonic capacity.*** Trait hedonic capacity was measured as in study 1 (Bernecker & Becker, 2021). Reliabilities for the full scale (α = 0.84) as well as the subscales was good (HS: α = 0.77, IT: α = 0.84).

***Alcohol use.*** First, participants indicated whether they had drank alcohol in the last three months (“yes”, “no”). Only participants that answered yes were included in the final sample. Alcohol use was measured with the German version of the Alcohol Use Identification Test (AUDIT: Saunders et al., 1993). Given that the study was carried out during the first Covid-19 lockdown, questions were framed accordingly. The test consists of ten questions (e.g., “Since the beginning of the lockdown, did you drink any alcoholic beverages?”). Answers for the first 8 questions were given on a 5-point scale. For each answer one could get 0 to 4 points. Answers for the final 2 questions were given on a 3-point scale (0, 2, 4 points). All points were summed, so that scores ranged from 0 to 40 (α = 0.79). Higher scores indicated more frequent alcohol use. Often also a categorical approach is taken, with scores from 0 to 7 considered low risk drinking, 8–15 hazardous and 16–19 harmful drinking, and above 20 high risk drinking (Babor et al., 2001).

***Drinking motives.*** To measure participants’ drinking motives we used the coping and enhancement subscales of the German version of the Drinking Motive Questionnaire Revised (DMQ-R; Cooper, 1994, Kuntsche et al., 2006). Five items measured coping motives (e.g., “How often since the beginning of the lockdown did you drink, to forget your worries?”). One item about drinking to reduce stress was added (6 items; α = 0.88). Five items measured enhancement motives (e.g., “…because it gives you a pleasant feeling?”; 5 items; α = 0.87). All questions were answered on a 5-point Likert scale, with higher scores indicating more endorsement (1 = *never* to 5 = *always*).

***Stress.*** Next, participants filled in the German version of the Perceived Stress Scale (Cohen et al., 1983, Reis et al., 2019). The scale consists of ten questions, such as “How often since the beginning of the lockdown … did you feel nervous or stressed”. Answers were given on a 5-point Likert scale ranging from 1 (never) to 5 (*very often*), and averaged. Higher scores indicate higher stress levels (α = 0.87).

#### 3.1.3 Analysis plan

We first computed bivariate correlations to check for overall associations between the variables of interest. To test H1, we conducted a linear regression regressing the AUDIT score on trait hedonic capacity (mean centered), controlling for gender (female = 1 male = −1). We added gender as a control variable to all analyses (preregistered as exploratory), because of its relevance in the context of drinking behavior (White, 2020).

To test H2, we conducted 2 linear regressions regressing the 1) coping and 2) enhancement motivation score on trait hedonic capacity (mean centered), controlling for gender (female = 1 male = −1).

Given we predicted null results for H1 (AUDIT) and H2 (enhancement motivation), we added two Bayesian linear regressions (not preregistered; in JASP, version 0.17.3, JASP Team, 2023) to test under which model (null model, THC, THC + gender) the data (H1: AUDIT and H2: enhancement) is most likely to occur. All models were selected to be of equal prior probability (uniform model prior).

For the first exploratory analysis testing the moderating role of stress, we conducted a linear regression regressing the AUDIT score on trait hedonic capacity (mean centered), stress (mean-centered) and the interaction of THC and stress, controlling for gender (female = 1 male = −1).

For the second exploratory analysis testing the relationship between coping motives and drinking behavior, we conducted a linear regression regressing the AUDIT score on coping motivation (mean centered), controlling for gender (female = 1 male = −1).

### 3.2 Results and discussion

Replicating the overall pattern of study 1, correlational analyses showed that THC was unrelated to alcohol use, but negatively related to coping motives (for both subscales; see Table 2). Coping motives in turn were positively related to alcohol use. The majority of participants fell in the low risk group (*n* = 259), followed by harmful drinking (*n* = 36), hazardous drinking (*n* = 6) and high risk groups (*n* = 1).

**Table 2:** Zero-Order Pearson Correlations for Trait Hedonic Capacity, Alcohol Use, Motivation to Drink (Coping, Enhancement) and Stress in Study 2.

Variable	*M*	*SD*	1	2	3	4	5	6
1. THC	3.12	0.67	–					
2. THC HS	3.56	0.68	0.82^***^ [0.78, 0.85]	–				
3. THC IT	3.22	0.88	−0.90^***^ [−0.92, −0.87]	−0.48^***^ [−0.56, −0.39]	–			
4. Alcohol use	4.38	3.97	−0.08 [−0.19, 0.03]	−0.06 [−0.17, 0.05]	0.08 [−0.04, 0.19]	–		
5. Coping	1.59	0.75	−0.29^***^ [−0.39, −0.18]	−0.29^***^ [−0.39, −0.19]	0.22^***^ [0.11, 0.32]	0.41^***^ [0.31, 0.50]	-	
6. Enhancement	2.59	1.04	0.05 [−0.07, 0.16]	0.01 [−0.11, 0.12]	−0.07 [−0.18, 0.05]	0.44^***^ [0.34, 0.53]	0.53^***^ [0.44, 0.61]	–
7. Stress	2.85	0.64	−0.54^***^ [−0.62, −0.46]	−0.47^***^ [−0.55, −0.38]	0.46^***^ [0.37, 0.55]	0.17^**^ [0.06, 0.28]	0.29^***^ [0.19, 0.39]	0.04 [−0.08, 0.15]

*Note*. THC = Trait hedonic capacity. THC HS = trait hedonic capacity subscale hedonic success. THC IT = trait hedonic capacity subscale intrusive thoughts. Numbers in brackets refer to 95 % confidence interval of the correlation coefficient. Significance levels: *** means *p* < .001; ** means *p* < .01; * means *p* < .05.

Given that the distribution of coping motive and AUDIT scores were skewed, we conducted the following robustness checks: we computed nonparametric Spearman correlations and ran additional regressions analyses with log-transformed AUDIT and coping scores. Those correlation and regression coefficients were largely identical with the ones reported here. Moreover, transformations had a positive effect on the degree to which assumptions were met for the regression analyses.

#### 3.2.1 Confirmatory analyses

**Fig. 1:**
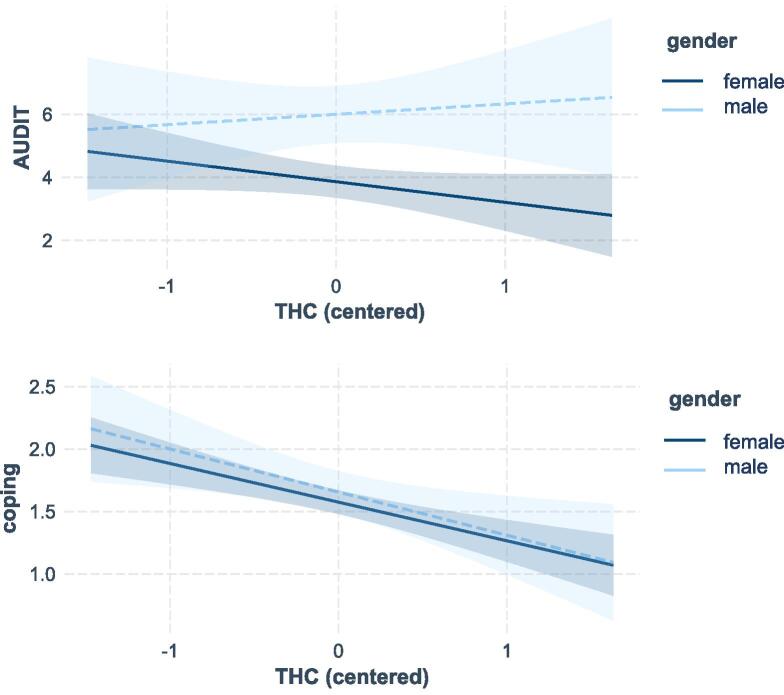
The Figure shows the Regression Coefficients for Trait Hedonic Capacity (THC) on Alcohol Use (AUDIT; Top Panel) and Coping Motives (Bottom Panel) for Female and Male Participants Separately (Based on a Regression Model Including the Interaction Term between THC and Gender) Note. Areas around the lines represent 95 % CI’s.

***Alcohol use.*** To test H1 we conducted a regression analysis predicting alcohol use (AUDIT sum score) with trait hedonic capacity (mean centered), controlling for gender (female = 1 male = −1). For all confirmatory analyses, effects remain the same when not including gender in the regression analyses. Moreover, additional analyses showed that gender never interacted with the focal predictor(s) in any of the confirmatory analyses. Effects are also similar across both THC subscales. All assumptions apart from the normality of residuals were met. Given our relatively large sample size, and considering the robustness checks mentioned above, our results should however still be valid (Schmidt & Finan, 2018). The model was significant, adjusted *R*^2^ = 0.05; *F*(2, 296) = 9.17, *p* < .001. As expected, trait hedonic capacity was unrelated to participants’ AUDIT score, *b* = −0.45, *SE* = 0.33; 95 % CI [−1.10, 0.21], β = −0.08, *p* =.183, whereas gender was, *b* = −1.07, *SE* = 0.26; 95 % CI [−1.58, −0.55], β = −0.23, *p* < .001. Male participants (*M* = 6.00; *SD* = 5.27) had higher AUDIT scores than female participants (*M* = 3.86; *SD* = 3.33; see Fig. 1). This finding is in line with general population data suggesting that males drink more alcohol than females (Eurostat, 2019, White, 2020).

The additional Bayesian linear regression (in JASP, version 0.17.3, JASP Team, 2023) supported the above findings. The model including only THC was the least likely one, P(M|data) < 0.001 and the model including only gender the most likely one P(M|data) = 0.72. The Bayes factor (BF_10_ = 0.001; model THC only) suggested that the observed data are 1000 (1/BF_10_) times more likely under the model containing gender only compared to under the model containing THC only. The data was also 2.63 times more likely under the model containing gender only compared to the model containing gender and THC (BF_10_ = 0.38). That suggests that THC is unrelated to participants’ AUDIT scores.

***Drinking motives.*** To test H2, we conducted another regression analysis with coping motivation as outcome variable, and THC (mean centered) as predictor, controlling for gender (female = 1 male = −1). All assumptions apart from the normality of residuals were met. The model explained a significant amount of variance, adjusted *R*^2^ = 0.08, *F*(2, 295) = 13.47, *p* < .001. This time THC was negatively related to coping motivation, *b* = −0.32, *SE* = 0.06; 95 % CI [−0.44, −0.20], β = −0.29, *p* < .001, and gender was not, *b* = −0.04, *SE* = 0.05; 95 % CI [−0.14, 0.06], β = −0.05, *p* =.407 (see Fig. 1).

A similar regression analysis showed that THC was unrelated to enhancement motivation (*t*s < 1.5, *p*s > 0.05). This was supported by an additional Bayesian linear regression analysis (equal prior odds) which showed the null model was the most likely one P(M|data) = 0.73. The Bayes factor (BF_10_ = 0.20; model THC only) suggested that the observed data are 5 (1/BF_10_) times more likely under the null model compared to under the model containing THC only. The data was also 25.64 times more likely under the null model compared to the model containing gender and THC (BF_10_ = 0.039). That suggests that THC is unrelated to participants’ enhancement motives.

#### 3.2.2 Exploratory analyses

**Fig. 2:**
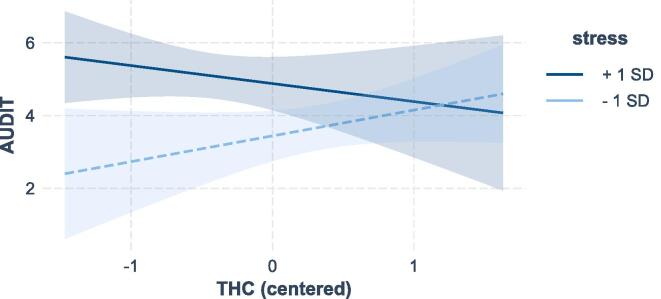
The Effect of Trait Hedonic Capacity on Alcohol Use (AUDIT) for Low (-1 SD) and High (+1 SD) Levels of StressNote. Areas around the lines represent 95 % CI’s.

We explored whether current stress levels would moderate the relationship between THC on alcohol use (controlling for gender). The model was significant, adjusted *R*^2^ = 0.09, *F*(2, 294) = 7.95, *p* < .001. The interaction was significant: *b* = −0.95, *SE* = 0.47; 95 % CI [−1.88, −0.02], β = −0.11, *p* =.045. Follow-up analyses showed that for people with low levels of THC (−1*SD*) stress levels were positively associated with alcohol use, *b* = 1.78, *SE* = 0.50, 95 % CI [0.80, 2.76], *p* =.004, whereas for high levels of THC (+1*SD*) they were not, *b* = 0.50, *SE* = 0.55, 95 % CI [−0.59, 1.59], *p* =.367 (see Fig. 2). With regards to drinking alcohol, people with high levels of trait hedonic capacity are thus less reactive to stress.

Finally, replicating earlier work we tested whether coping motives would be related to alcohol use (controlling for gender). The model was significant, adjusted *R*^2^ = 0.21, *F*(2, 295) = 39.87, *p* < .001. Coping motives were positively related to AUDIT scores, *b* = 2.14, *SE* = 0.28; 95 % CI [1.59, 2.68], β = 0.40, *p* < .001. This suggests that drinking for coping motives is related to increased alcohol intake.

## 4 General discussion

Two studies consistently showed that in a sample of individuals predominantly at low risk of harmful alcohol use, trait hedonic capacity was unrelated to alcohol use (H1), but negatively related to coping motives (H2). Given that coping motives are the motive most strongly related to harmful and problematic alcohol use (Cooper et al., 2015), it is important to undcover factors related to it. Additionally, we replicated a finding in the general public that males drink more alcohol than females (Eurostat, 2019, White, 2020). Moreover, the exploratory moderation analysis in study 2 suggested that THC *was* related to alcohol use when taking into account people’s stress levels: people with low THC showed increased alcohol use with increasing levels of stress. People high in THC did not show such stress reactivity. That suggests that high levels of trait hedonic capacity may protect people from drinking as a response to stress. Taken together, those findings are in line with previous work showing that people’s capacity to experience pleasure plays a significant role in understanding motivations for alcohol consumption (Destoop et al., 2019, Garland, 2021, Leventhal et al., 2010, Meehl, 1975, Snaith, 1993). They also extend previous work by suggesting that the link between hedonic capacity and alcohol use in a nonclinical sample may be indirect, through coping motives or through stress responsiveness. People low in THC are more motivated to drink out of coping motives and more likely to drink as a response to stress.

Our findings align with current developments in addiction treatment, which acknowledge that increasing people’s hedonic capacity could prevent harmful alcohol use as well as support treatment effectiveness. Stress is one vulnerability factor in developing a substance dependency (Berridge & Robinson, 2016). Our data suggests that people high in trait hedonic capacity are less at risk to cope with stress using alcohol which should work as protective factor. One example of a preventive approach incorporating the idea that high hedonic success might protect people from consuming substances is the Icelandic Model of Preventing Adolescent Substance Use (e.g., Kristjansson et al., 2020). The model takes a systemic approach to adolescent harmful alcohol use and includes access to ‘high quality leisure time’ (e.g., sport, music, drama) as one of the four protective factors in their model (besides family, peer group and school environment). The Icelandic approach was successful, alcohol use and drunkenness (in the period of 30 days before measurement) decreased from 39 % and 29 % in 1997 to 7 % and 3 % in 2014 (Kristjansson et al., 2015).

Of course, it is difficult to estimate the unique contribution of leisure activities to those effects. But by providing those opportunities young people can find out what they like, and what it is to enjoy leisure activities. Both are necessary for learning that there are multiple ways through which one can upregulate positive affect and downregulate negative affect besides substance use. Having more means available to achieve those regulatory goals is important, because then their reinforcing properties spread across multiple means rather than just one. That means that all means carry a fraction of the reinforcement potential rather than one carrying all (Köpetz et al., 2013). Addiction prevention or treatment programs should, therefore, consider including the ‘restructuring of rewards’ (Garland et al., 2019, Garland, 2021), which refers to helping people derive pleasure and enjoyment from *natural* rather than drug-related reinforcers (e.g., food, leisure activities). One intervention that pioneers that novel approach is mindfulness-oriented recovery enhancement (MORE), which includes savoring as one of its key elements (Bryan et al., 2022, Garland et al., 2019, Garland, 2021). Other interventions taking a related approach use neurofeedback to teach drug users regulate activity in reward related brain areas (i.e., ventral tegmental area and substantia nigra; Kirschner et al., 2018) or train them to vividly imagine the pursuit of naturally reinforcing goals (May et al., 2015). Based on our findings, these programs might be especially effective in people low in hedonic capacity and they might work, because they increase people’s hedonic capacity (see Garland et al., 2023). Taken together, current approaches to addiction prevention and intervention started to acknowledge the importance of supporting people in finding pleasure and enjoyment in natural reinforcers and thereby possibly increase their hedonic capacity.

There are also limitations to our findings. First, given the cross-sectional nature of our data we cannot draw causal conclusions about whether differences in THC cause differences in motivation to drink. Neither can we conduct a meaningful mediation analysis testing whether trait hedonic capacity might indirectly affect drinking behavior through motivation (Fiedler et al., 2011). We acknowledge that even though one could argue that a relatively stable trait is likely to influence motivations and thereby behavior, there could be other third unknown factors causing co-variation in all three of our variables. Moreover, the interplay between traits, motives and behavior is obviously more dynamic than one-directional. Nevertheless, we are convinced that our results still add a valuable piece to our understanding of that dynamic, especially given the growing interest in how hedonic capacity or hedonic experiences are related to alcohol consumption (Garland et al., 2023, Garland, 2021). More research is needed to look at the relationships between trait hedonic capacity, drinking motives, and alcohol use with a prospective design.

Second, even though the purpose of the study was to look at self-regulatory processes in a non-clinical sample, we cannot rule out that variations in depressive symptoms (i.e., anhedonia), or other third variables, have influenced our results. Previous work has established a general link between depressive symptoms and coping motives in the context of drinking behavior (O’Hare & Shen, 2012), but the specific relationships vary across studies, with some studies finding no relationship (Grazioli et al., 2018) and some effects for especially low levels of depression (Kenney et al., 2017). Moreover, whereas trait hedonic capacity is associated with physical symptoms of depression, it is unrelated to the more affect-based measure of anhedonia (Bernecker & Becker, 2021). Future studies should, therefore, include measures of depressive symptoms to help elucidate the interrelation between the difference variables.

Third, it is surprising that there was no relationship between trait hedonic capacity and enhancement motives in either of the studies. One could have speculated that people with low hedonic capacity might be more motived to use alcohol also for enhancement because they lack the capacity to generate the hedonic experience themselves. Our finding, however, suggest otherwise and fit other recent findings showing that people with low hedonic capacity are generally less motivated and likely to approach hedonic experiences in general (Bernecker et al., 2023). Finally, study 2 was conducted during the first Covid-19 lockdown. Some studies suggest that people had different drinking habits during the lockdown, with some drinking more and some less (Koopmann et al., 2020, Koopmann et al., 2021, Niedzwiedz et al., 2021). But given that we find the same pattern also in study 1, which was conducted before the lockdown, we remain confident that the main confirmatory findings are reliable.

## 5 Conclusion

Two studies (one preregistered) showed that people with low trait hedonic capacity have a higher motivation to drink out of coping reasons. We also show that people low in trait hedonic capacity might be more likely to drink as a response to stress. Our findings complement existing work in the clinical domain, and emphasizes the importance of acknowledging the role of hedonic capacity in prevention and intervention designs.

## CRediT authorship contribution statement

**Daniela Becker:** Writing – review & editing, Writing – original draft, Supervision, Project administration, Methodology, Investigation, Formal analysis, Data curation, Conceptualization. **Katharina Bernecker:** Writing – review & editing, Methodology, Investigation, Data curation, Conceptualization.

## Declaration of competing interest

The authors declare that they have no known competing financial interests or personal relationships that could have appeared to influence the work reported in this paper.

## Data Availability

The data is available on the Open Science Framework (https://osf.io/bjp8a/).
